# Vitamin D Levels in Patients with Overlap Syndrome, Is It Associated with Disease Severity?

**DOI:** 10.3390/jpm12101693

**Published:** 2022-10-11

**Authors:** Kostas Archontogeorgis, Athanasios Voulgaris, Evangelia Nena, Athanasios Zissimopoulos, Izolde Bouloukaki, Sophia E. Schiza, Paschalis Steiropoulos

**Affiliations:** 1 MSc Program in Sleep Medicine, Medical School, Democritus University of Thrace, 68100 Alexandroupolis, Greece; 2 Department of Pneumonology, Medical School, Democritus University of Thrace, 68100 Alexandroupolis, Greece; 3 Laboratory of Social Medicine, Medical School, Democritus University of Thrace, 68100 Alexandroupolis, Greece; 4 Laboratory of Nuclear Medicine, Medical School, Democritus University of Thrace, 68100 Alexandroupolis, Greece; 5 Sleep Disorders Unit, Department of Respiratory Medicine, Medical School, University of Crete, 71110 Heraklion, Greece

**Keywords:** chronic obstructive pulmonary disease, obstructive sleep apnea, overlap syndrome, vitamin D

## Abstract

Background: The coexistence of chronic obstructive pulmonary disease (COPD) and obstructive sleep apnea (OSA) has been defined as overlap syndrome (OVS). Recently, a link between OSA, COPD and Vitamin D (Vit D) serum concentration was reported, however, evidence regarding Vit D status in patients with OVS is scarce. The aim of the present study was to evaluate Vit D serum levels and to explore the association of those levels with anthropometric, pulmonary function and sleep parameters in patients with OVS. Methods: Vit D serum levels were measured in patients diagnosed with OVS, as confirmed by overnight polysomnography and pulmonary function testing. Results: A total of 90 patients (79 males and 11 females) were included in the analysis. The patients were divided into three groups matched for age, gender, and BMI: the control group that included 30 patients (27 males and 3 females), the OSA group that included 30 patients (26 males and 4 females), and the OVS group that included 30 patients (26 males and 4 females). Patients with OVS exhibited decreased serum 25(OH)D levels compared with OSA patients and controls (14.5 vs. 18.6 vs. 21.6 ng/mL, *p* < 0.001). In the OVS group, multiple linear regression analysis identified AHI and FEV_1_, as predictors of serum 25(OH)D levels (*p* = 0.041 and *p* = 0.038, respectively). Conclusions: Lower Vit D levels have been observed in patients with OVS compared with OSA patients and non-apneic controls, indicating an increased risk of hypovitaminosis D in this population which might be associated with disease severity.

## 1. Introduction

Chronic obstructive pulmonary disease (COPD) and obstructive sleep apnea (OSA) are both highly prevalent pulmonary diseases [1,2]. COPD affects approximately 11.7% of the global adult population, and OSA is estimated to affect 10–17% men and 3–9% women [1,2]. The coexistence of the two conditions has been defined as “overlap syndrome (OVS)”, a distinct clinical syndrome, which may be different to the simple aggregate of OSA and COPD [3]. Epidemiological studies indicate an OVS prevalence of approximately 1% among adult males [4]. Both COPD and OSA share common risk factors such as tobacco smoking, and are associated with systemic inflammation and oxidative stress [4]. Additionally, patients with OVS are known to exhibit a more profound hypoxia during sleep compared with patients with COPD or OSA alone, further contributing to inflammatory activation and the potential development and progression of cardiovascular disease and other comorbidities [5,6]. OSA and COPD have also been associated with an increased risk of endocrinal and metabolic conditions, namely insulin resistance, depression of the somatotropic axis, hypogonadism, imbalance of the adrenal axis, bone loss and hypothyroidism [7,8,9].

Recently, a link between OSA, COPD and Vitamin D serum concentration was reported in the literature [10,11,12]. Vitamin D (Vit D) is a fat-soluble vitamin that can be found in some foods and is synthesized in the skin after exposure to sunlight [13]. The classical function of Vit D is the regulation of bone metabolism; however, accumulating evidence suggests that it also possesses anti-inflammatory and immune-modulating properties [13]. Serum 25-hydroxyvitamin D (25(OH)D) concentration is recommended as the best indicator of Vit D status [13]. Vit D insufficiency has exponentially increased over the years, with its prevalence estimated between 13% and 40.4% in European countries, depending on the definition of Vit D insufficiency used [14]. Decreased serum 25(OH)D has been associated with a variety of pulmonary diseases, including respiratory infections, asthma and cancer [13]. Recently, OSA has been associated with hypovitaminosis D, and adequate use of CPAP is likely to augment Vit D levels [10,15]. Similarly, decreased serum 25(OH)D levels have been described in COPD patients, while a protective role against exacerbations has been attributed to Vit D supplementation [11,16]. However, evidence regarding Vit D status in patients with OVS is scarce. Therefore, the aim of the present study is to evaluate Vit D serum levels and to explore the association of those levels with anthropometric, pulmonary function and sleep parameters in patients with OVS.

## 2. Methods

### 2.1. Study Population

The present study protocol was approved by the Alexandroupolis University General Hospital Ethics Committee and all performances were carried out in accordance with the Helsinki Declaration of Human Rights [17]. Participants who enrolled between January of 2019 and December of 2020 from the sleep laboratory of our institution were referred for evaluation of suspected sleep-disordered breathing. Informed consent was provided prior to each participant’s enrollment.

Patients diagnosed with both OSA and COPD, as confirmed by overnight polysomnography and pulmonary function testing were included in the study. Exclusion criteria were as follows: refusal to participate, previously diagnosed OSA under continuous positive airway pressure (CPAP) therapy or evidence of exclusively central apneas documented in polysomnography, Vit D supplementation, conditions known to affect calcium, phosphorus and Vit D metabolism and absorption, serious heart diseases, chronic kidney or hepatic disease, cancer, inflammatory diseases, severe cognitive or psychiatric disorders, or any respiratory disorder other than COPD.

### 2.2. Study Variables

Information on previous medical history, current medication use (with an emphasis on Vit D supplementation) and tobacco smoking were obtained from all participants. Anthropometrical data including age, sex, neck, waist and hip circumference were also recorded, and a waist-to-hip ratio (WHR) was calculated. Body mass index (BMI) was calculated using the following formula: BMI = Weight (in kilograms)/Height (in meters)^2^. Subjective sleepiness was assessed using the Greek version of the Epworth Sleepiness Scale (ESS), which assesses the tendency to fall asleep during eight typical daytime situations [18]. A score >10 was considered indicative of excessive daytime sleepiness [18].

### 2.3. Polysomnography

All participants underwent an overnight, in-laboratory supervised polysomnography from 22:00 to 06:00 and the data were recorded on a computer system (Alice^®^ 4, Philips Respironics, Murrysville, PA, USA). In each patient, electroencephalogram, electrooculogram, electromyogram (submental and bilateral tibial), and electrocardiogram were recorded. Airflow was detected using combined oronasal thermal sensors. Additionally, continuous pulse oximetry was performed, and impedance belts were used to assess the thoracic and abdomen respiratory effort. Respiratory events (apneas and hypopneas) and electroencephalogram recordings were manually scored according to international guidelines [19]. Apnea was defined as a cessation of airflow or ≥90% of airflow reduction for at least 10 s [19]. Obstructive apneas were defined based on the presence of thoracic efforts. Hypopnea was defined as ≥50% decrement in airflow for at least 10 s in combination with oxyhemoglobin desaturation of at least 4% [19]. The apnea-hypopnea index (AHI) was calculated as the average number of apneas and hypopneas per hour of recorded sleep time [19]. A diagnosis of OSA was made when the AHI in the recorded study was >5 events/h of sleep accompanied by related symptoms (sleepiness, snoring, witnessed apneas) [20]. Subjects with AHI <5/h of sleep were considered as controls.

### 2.4. Pulmonary Function Testing

Pulmonary function testing (Screenmate, Erich Jaeger GmbH & Co., Hochberg, Germany) and arterial blood gasses analysis were performed the day before PSG. Forced expiratory volume in the first second (FEV_1_) and forced vital capacity (FVC) were measured and the FEV_1_/FVC ratio was calculated. The diagnosis of COPD was based on the presence of persistent airflow limitation, as defined by a post-bronchodilator FEV_1_/FVC ratio of less than 70%, associated with symptoms such as dyspnea, cough and/or sputum production and a compatible history of exposure to risk factors [21].

### 2.5. Blood Samples and Analysis

All participants provided a fasting blood sample upon awakening the morning after polysomnography. Blood was then centrifuged (3000 rpm for 10 min) and the serum obtained was conserved frozen at −80 °C for future analysis. Serum 25(OH)D was measured using a commercial radioimmunoassay kit (DiaSorin, Stillwater, MN, USA). Biochemical examinations regarding renal, hepatic function, as well as lipidemic profile were also performed.

### 2.6. Statistical Analysis

All analyses were performed using the IBM Statistical Package for Social Sciences version 17.0 (SPSS Inc., Chicago, IL, USA, 2008). Study participants were divided into three groups matched for age, gender and BMI, as controls, OSA and OVS. All variables were presented as median (25th–75th percentile) for continuous variables and proportions for categorical variables. Comparison of baseline characteristics among groups was performed by a chi-square test for categorical variables, or a one-way ANOVA for continuous variables. Post -hoc analysis was performed using the Tukey’s post-hoc test when homogeneity of variances was assumed, and the Games-Howell’s post-hoc test when the assumption of homogeneity of variances was violated. Multiple linear regression analysis was used to test the association of serum 25(OH)D levels with sleep, anthropometric and respiratory function parameters.

## 3. Results

A total of 90 patients (79 males and 11 females) were included in the analysis. In general, participants were middle-aged (median age 57 years) and obese (median BMI 36.2 kg/m^2^) with a median value of serum 25(OH)D concentration of 18.1 (13.4–24.4) ng/mL. The sample was divided into three groups matched for age, gender and BMI: the control group that included 30 subjects (27 males and 3 females), the OSA group that included 30 patients (26 males and 4 females) and the OVS group that included 30 patients (26 males and 4 females). Neck and waist circumference differed between the groups (*p* = 0.005 and *p* = 0.023, respectively), and patients with OVS were more likely to be current or ex-smokers. The baseline characteristics of the three groups are shown in Table 1.

Regarding sleep parameters, AHI was similar between OSA and OVS patients (40.2 vs. 37.1 events/h of sleep, *p* > 0.05). Patients with OVS exhibited the worst oxygenation during sleep compared with OSA patients, as assessed by the average oxygen saturation during sleep (90.8% vs. 92%, *p* < 0.05) and the percentage of time with oxygen saturation during sleep < 90% (27.3% vs. 19.7%, *p* < 0.05). No difference was noted between the groups in terms of daytime sleepiness, as expressed by the Epworth sleepiness scale score (*p* = 0.106). Comparisons of sleep parameters between the groups are presented in Table 2.

**Figure 1:**
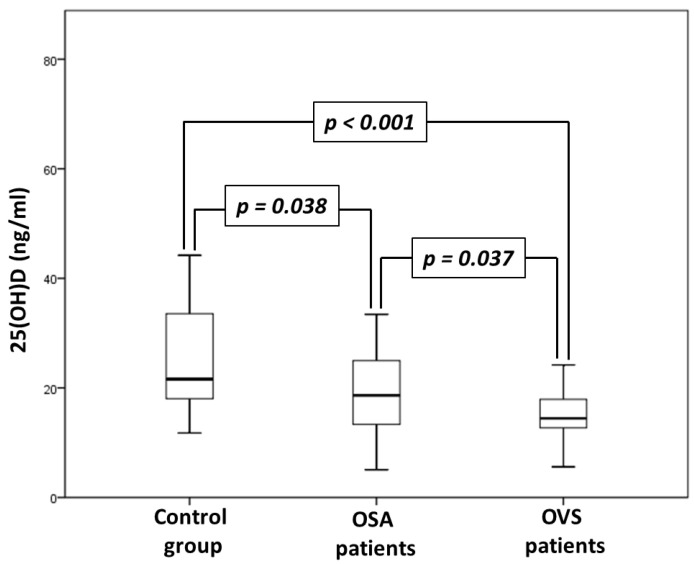
Comparison of 25(OH)D serum levels between control, obstructive sleep apnea syndrome (OSA) and overlap syndrome (OVS) groups.

In the OVS group, the median COPD assessment test (CAT) score was 9 (5–14.3). According to the GOLD classification, the OVS group included 7 patients with mild, 16 patients with moderate, 6 patients with severe and 1 patient with very severe airflow limitation [21]. Patients with OVS had a lower FEV_1_ compared with the OSA patients and control patients (64.7 vs. 89.9 vs. 105.5%, respectively, *p* < 0.001) and presented increased PaCO_2_ during wake in respect to both the OSA and control patients (46.5 vs. 41 vs. 40.5 mmHg, respectively, *p* < 0.001). Patients with OVS also exhibited decreased serum 25(OH)D levels compared with the OSA patients and control patients (14.5 vs. 18.6 vs. 21.6 ng/mL, *p* < 0.001) (Figure 1). Comparisons of measurements and laboratory analyses between the groups are presented in Table 3.

In order to explore the factors predicting serum 25(OH)D levels, a regression model was created with 25(OH)D serum levels used as a dependent variable. Anthropometric (age, gender, BMI), pulmonary function parameters (FEV_1_, FVC and CAT score), and sleep parameters (AHI, average and minimum saturation during sleep, time spent with SaO_2_ < 90%, arousal index and ESS score) were used as independent variables. Multiple linear regression identified that serum 25(OH)D levels could be predicted by AHI (β = −0.758, *p* = 0.041, 95% CI: −0.282–−0.006) and FEV_1_ (β = 0.698, *p* = 0.038, 95% CI: 0.014–0.410).

## 4. Discussion

The OVS is characterized by the co-existence of COPD and OSA, both conditions that seem to have an adverse effect on calcium homeostasis. In this study, we found that 25(OH)D serum levels were decreased in patients with OVS, compared with both OSA patients and non-apneic controls. Additionally, AHI and FEV_1_ were independently associated with 25(OH)D serum levels in OVS patients.

The definition of Vit D status in OSA patients remained controversial for a long time, with some studies reporting decreased Vit D levels in those patients while others concluded otherwise [22,23,24,25]. A meta-analysis conducted by Neighbors et al., [26] including 14 studies with 1513 controls and 3424 OSA patients, reported decreased Vit D serum levels in the latter, with Vit D insufficiency being incrementally exacerbated with increasing severity of OSA (mean differences were −2.7% for mild OSA, −10.1% for moderate OSA and −17.4% for severe OSA). Similarly, results from a more recent meta-analysis conducted by Li et al. [10] showed decreased Vit D levels only in patients with moderate (*p* = 0.002) and severe (*p* < 0.0001) OSA compared with non-apneic controls. OSA treatment with CPAP seems to possess a beneficial effect on Vit D serum levels. Liguori et al. found increased levels of 25(OH)D after 7 nights of CPAP therapy in male OSA patients, and after 1 year of treatment mainly in obese OSA patients [27,28]. Similar conclusions were reached in a randomized sham-controlled trial, where 24 weeks of CPAP treatment improved Vit D serum levels (*p* = 0.045) in patients with severe OSA [29]. In the present study, Vit D serum levels were strongly associated with AHI. These results are in line with those from previous studies. In a study that included 139 OSA patients and 30 controls, OSA patients had lower 25(OH)D levels compared with controls (17.8 ± 7.8 vs. 23.9 ± 12.4 ng/mL respectively, *p* = 0.019) and there was a significant inverse association between AHI and 25(OH)D levels (r = −0.187, *p* = 0.045) [22]. Similarly, in another study that included 75 OSA patients and 31 controls, serum 25(OH)D levels decreased with OSA severity (*p* = 0.003) and were negatively associated with AHI (r = −0.40, *p* = 0.0001) [30]. Overall, current evidence suggests that hypovitaminosis D is frequent among OSA patients. Multiple factors such as BMI, gender and disease severity may play a regulatory role in this relationship.

Previous studies have shown that, compared with controls, patients with COPD frequently presented with decreased Vit D levels, with a prevalence that ranged between 33% and 77% according to disease severity [31,32,33]. In a recent study that included 1609 COPD patients, Vit D deficiency was present in 21% and was associated with a 4.11% decrease in predicted FEV_1_ at enrollment (95% CI: −6.90% to −1.34% predicted FEV_1_, *p* = 0.004), a 1.27% predicted greater rate of FEV_1_ decline after 1 year (95% CI: −2.32% to −0.22% predicted/year, *p* = 0.02), and increased odds of any COPD exacerbation in the prior year (OR: 1.32, 95% CI: 1.00–1.74, *p* = 0.049) [11]. Studies evaluating the effect of Vit D supplementation in lung function produced contradictory results. In a meta-analysis that included 8 studies and 687 COPD patients, Vit D treatment resulted in no significant improvements in FEV_1_ (*p* = 0.144), FVC (*p* = 0.299), and FEV_1_/FVC (*p* = 0.995) in COPD patients [34]. On the other hand, another meta-analysis that included 25 studies involving 2670 COPD patients concluded that Vit D supplementation was significantly associated with FEV_1_ (*p* < 0.01), FEV_1_/FVC (*p* < 0.01), exacerbations (*p* < 0.01), sputum volume (*p* < 0.01), 6-min walk distance (*p* = 0.02) and CAT score (*p* < 0.01) [35].

Vit D insufficiency in OVS is multifactorial and represents the result of the interaction of individual conditions present in COPD and OSA. In particular, altered cutaneous synthesis due to aging, toxic smoke effects and reduced sun exposure because of disability to perform outdoor activities, sequestration in the adipose tissue, increased Vit D catabolism caused by glucocorticoid therapy (together with impaired liver and renal activation and reduced gastrointestinal absorption) are all factors known to reduce Vit D serum levels in COPD patients [36]. Reduced outdoor activities due to exertional dyspnea may also negatively affect Vit D cutaneous synthesis. In addition, low 25(OH)D levels in COPD correlate with genetic variants of the Vit D-binding gene [32]. Conversely, OSA may represent a risk factor for Vit D insufficiency. Lack of outdoor activity due to obesity and excessive daytime sleepiness might result in reduced Vit D synthesis because of insufficient sun exposure [37]. Moreover, Vit D is stored in fat tissue, thus reducing the release of Vit D into the circulation and decreasing its bioavailability [37]. Finally, Vit D receptor gene variations affect both 25(OH)D serum levels and disease susceptibility in OSA [38].

A previous study has shown that OVS patients had a similar Vit D status compared to the controls, OSA, or COPD patients and Vit D deficiency as associated with overall mortality among groups [39]. Diagnosis of Vit D insufficiency and correction of Vit D status has several clinical implications in patients with both OSA and COPD. Notably, hypovitaminosis D is highly prevalent in COPD patients and has been associated with worse lung function, increased symptoms and rate of exacerbations, and worse prognosis [11,35]. Moreover, decreased Vit D serum levels were related to COPD severity, and acute exacerbations may be prevented with adequate Vit D supplementation [16,40]. Finally, there is evidence to suggest that a normal Vit D status could improve prognosis in patients with COPD exacerbations caused by respiratory tract infections [41]. Serum 25(OH)D levels were decreased in OSA patients compared with controls, and have been associated with disease severity [10]. On the other hand, with increasing OSA severity serum 25(OH)D levels further decreased, this suggests that serum 25(OH)D might be a risk factor for OSA [10]. Finally, markers of inflammation and HOMA-IR were significantly decreased after Vit D supplementation in a small group of patients with mild OSA [42].

The relationship between an adequate Vit D status and cardiovascular risk is controversial, with data suggesting a negative association between Vit D levels and the risk of cardiovascular disease. Whereas, other studies failed to demonstrate an association between Vit D serum levels and a reduced risk for adverse cardiovascular events [43,44]. In a study conducted in Southern Italy that included 451 participants, the Vit D level did not correlate with cardiovascular risk, as assessed using the Framingham cardiovascular risk charts [45]. On the contrary, the parathyroid hormone was in direct correlation (*p* < 0.001) with cardiovascular risk, and increased parathyroid hormone levels identified a population with a higher risk for cardiovascular events (*p* < 0.001) [45]. Results from this study suggest increased parathyroid hormone levels might be a better predictor of cardiovascular risk in patients with hypovitaminosis D.

Nonetheless, the present study has limitations. First, the study was cross-sectional in design, and consequently, it is not possible to infer any causal relationships. Second, the sample of OVS patients included was rather small and was not representative of the female population. This is largely due to the low prevalence of OVS in the general population and to the disparity in gender-specific estimates of COPD and OSA prevalence [4,46,47]. Thus, larger scale studies are necessary to better assess Vit D status in OVS patients. Third, 25(OH)D was not deseasonalized and the parathyroid hormone was not measured. However, all participants were Caucasian, living in a single region of northern Greece, a relatively small geographical area, with no variations in latitude and with similar conditions regarding cloudiness and air pollution. Moreover, participants had no variations regarding skin pigmentation and in their way of dressing, had similar dietary habits, similar sun exposure and sun-bathing behavior. Furthermore, OVS patients were middle-aged, so caution is warranted in order to extrapolate this data to elderly OVS patients. Finally, only one patient with very a severe air-flow limitation was included in the OVS group. However, it has been shown that Vit D levels decrease with increasing air-flow limitation severity [11]. An analysis of sub-populations based on disease severity in the OSA group was not performed due to the limited sample size.

## 5. Conclusions

In conclusion, lower Vit D levels have been observed in patients with OVS compared with OSA patients and non-apneic controls, indicating an increased risk of hypovitaminosis D in this population. This is expected since both the components of OVS, COPD and OSA are also associated with decreased Vit D levels. Given the protective role of Vit D against FEV_1_ decline and COPD exacerbations, as well as the association of low Vit D levels with cardiovascular and metabolic diseases, screening of OVS patients for Vit D might be beneficial in the management of this population of patients.

## Figures and Tables

**Table 1:** Comparison of anthropometric characteristics between control, obstructive sleep apnea (OSA) and overlap syndrome (OVS) groups.

	Control Group (*n* = 30)	Patients with OSA (*n* = 30)	Patients with OVS (*n* = 30)	*p*
Gender (males/females)	27/3	26/4	26/4	0.902
Age (years)	56 (48.8–64.3)	56 (52.8–65)	60 (54.8–67.3)	0.386
Neck circumference (cm)	41 (38–45.5)	46 (43.3–48.8) *	45 (44–50) *	0.005
Waist circumference (cm)	119 (104–131.5)	122 (119–129)	130 (119–135.5) *	0.023
Hip circumference (cm)	119 (107.5–125.5)	117 (112.3–122.8)	119 (110–123.5)	0.554
WHR	1.01 (0.94–1.05)	1.03 (1–1.08)	1.08 (1.04–1.11) *	0.040
BMI (kg/m^2^)	33.6 (29.9–40.2)	36.9 (34.5–41.6)	36.5 (32–42)	0.105
Tobacco smoking				
Non-smokers	36.7%	30%	0% **^, #^	0.001
Ex-smokers	36.7%	13.3% *	40% ^#^	0.049
Current smokers	26.7%	56.7% *	60% *	0.017

Abbreviations: BMI, body mass index; WHR, waist to hip ratio; *: *p* < 0.05 compared with control group; **: *p* < 0.001 compared with control group; #: *p* < 0.05 compared with OSA group.

**Table 2:** Comparison of sleep characteristics between control, obstructive sleep apnea syndrome (OSA) and overlap syndrome (OVS) groups.

	Control Group (*n* = 30)	Patients with OSA (*n* = 30)	Patients with OVS (*n* = 30)	*p*
TST (min)	326.3 (280.8–358.5)	310.1 (260.8–346.3)	320.5 (278.3–360.8)	0.843
N1 (%)	10 (5.6–17.2)	11.4 (6.6–23.4)	11 (4.8–18.2)	0.191
N2 (%)	62.2 (56.9–71)	66.7 (56.2–77.3)	66.4 (60.7–71.4)	0.676
N3 (%)	11.7 (4.4–20.5)	7 (2.1–15.8)	9.9 (6.6–19.1)	0.391
REM (%)	13.5 (4.9–15.1)	6.2 (1.2–13.7) *	4.1 (0.3–12.3) *	0.011
Sleep efficiency (%)	84.6 (75–91.9)	86.4 (74.2–91.9)	83.2 (72.4–92.2)	0.871
Arousal index	14.4 (9.1–18.2	36.5 (21.5–47.4) **	26.1 (10.3–44.9) *	<0.001
AHI (events/h)	2.9 (1.5–4)	40.2 (22.1–70) **	37.1 (18–62.6) **	<0.001
Aver SaO_2_ (%)	94.3 (93.8–95.3)	92(89–93) **	90.8 (84.9–92.1) **^,#^	<0.001
Min SaO_2_ (%)	88 (84–89)	74.5 (71.8–83.8) **	74.5 (63.5–81.3) **^,#^	<0.001
T < 90% (%)	0.1 (0–0.4)	19.7 (3.8–39.4) *	27.3 (7.7–83.1) **^,#^	<0.001
ESS score	8 (4.8–12.3)	11.5 (7.8–15.3)	9 (6–13.8)	0.106

Abbreviations: AHI, apnea hypopnea index; Aver SaO_2_, average oxyhemoglobin saturation; ESS, Epworth sleepiness scale; Min SaO_2_, minimum oxyhemoglobin saturation; N1, sleep stage 1; N2, sleep stage 2; N3, sleep stage 3; REM, rapid eye movement; TST, total sleep time; T < 90%, time with oxyhemoglobin saturation <90%; *: *p* < 0.05 compared with control group; **: *p* < 0.001 compared with control group; #: *p* < 0.05 compared with OSA group.

**Table 3:** Comparison of measurements and laboratory analyses between control, obstructive sleep apnea syndrome (OSA) and overlap syndrome (OVS) groups.

	Control Group (*n* = 30)	Patients with OSA (*n* = 30)	Patients with OVS (*n* = 30)	*p*
FEV_1_ (% predicted)	105.5 (91.3–113.8)	89.9 (74.6–100.3)	64.7 (48.8–76) **^, ##^	<0.001
FVC (% predicted)	102.5 (87.5–111.4)	85.6 (71–97.3) *	77.9 (62.4–89.1) **	<0.001
FEV_1_/FVC (%)	83 (80.4–87.8)	84.6 (79.1–90.3)	67.1 (62.5–69.2) **^, ##^	<0.001
pO_2_ (mmHg)	79 (73.5–84.3)	75 (66.8–79.5) *	67 (60.8–76) **	<0.001
pCO_2_ (mmHg)	40.5 (37–43)	41 (37–44.5)	46.5 (42–52) **^, #^	<0.001
Glucose (mg/dL)	95 (81–112.5)	112.5 (96,5–151.3)	112.5 (95.3–129.3)	0.080
Creatinine (mg/dL)	0.8 (0.75–1)	0.9 (0.8–1.1)	0.9 (0.8–1.1)	0.707
SGOT (U/L)	20 (17.5–22.3)	22 (17.3–27)	20 (17–24)	0.296
SGPT (U/L)	19.5 (16.8–28)	24 (18–30.5)	21.5 (16.8–26.3)	0.624
Cholesterol(mg/dL)	207 (182–258)	210.5 (162.5–242.8)	185.5 (149–214.5)	0.154
Triglycerides (mg/dL)	158 (117.5–214.3)	158 (120.5–190)	156.5 (117–244.8)	0.166
LDL-C (mg/dL)	128.2 (101.9–159.4)	124.2 (95.4–156.1)	96.1 (77.3–126.2) *	0.030
HDL-C (mg/dL)	46 (41.5–56)	44.5 (35.8–52.5)	50 (43–56.3)	0.411
25(OH)D (ng/mL)	21.6 (17.8–33.6)	18.6 (13.2–25.2) *	14.5 (12.3–17.9) **^, #^	<0.001

Abbreviations: FEV_1_, forced expiratory volume in first second; FVC, forced vital capacity; HDL-C, high density lipoprotein cholesterol; LDL-C, low density lipoprotein cholesterol; pCO_2_, carbon dioxide partial pressure; pO_2_, oxygen partial pressure; SGOT, serum glutamate-oxaloacetate transaminase; SGPT, serum glutamate-pyruvate transaminase; 25(OH)D, 25-hydroxyvitamin D; *: *p* < 0.05 compared with control group; **: *p* < 0.001 compared with control group; #: *p* < 0.05 compared with OSA group; ##: *p* < 0.001 compared with OSA group.

## Data Availability

Not applicable.
